# Full-scale simultaneous partial nitrification, anammox, and denitrification for the efficient treatment of carbon and nitrogen in low-C/N wastewater

**DOI:** 10.1016/j.wroa.2024.100288

**Published:** 2024-12-02

**Authors:** Xi Cao, Tianqi Liu, Xiang Li, Yong Huang, Qin Nie, Ming Li

**Affiliations:** a School of Environmental Science and Engineering, Suzhou University of Science and Technology, Suzhou, 215009, China; b Jiangsu Collaborative Innovation Center of Technology and Material of Water Treatment, Suzhou University of Science and Technology, Suzhou, 215009, China; c Suzhou Tianjun Environmental Technology Limited Company, Suzhou, 215011, China; d Suzhou Mengze Environmental Engineering Limited Company, Suzhou, 215100, China; e Qinhuangdao Huaheng Biological Limited Company, Qinhuangdao, 066000, China

**Keywords:** SNAD, C/N, Anammox, Denitrification, Partial nitrification

## Abstract

A full-scale simultaneous partial nitrification, anaerobic ammonia oxidation (anammox), and denitrification (SNAD) reactor was initiated to address the problem of high energy consumption for the treatment of low C/N wastewater. The SNAD system achieved a nitrogen removal rate of 0.9 kg/(m^3^·d) at an influent NH₄^+^–N concentration of 500 mg/L after 450 days of stable operation. Partial nitrification was achieved by maintaining free ammonia levels at 0.8 ± 0.3 mg/L and dissolved oxygen concentrations between 0.3 mg/L and 1.2 mg/L, which resulted in synergistic nitrogen removal, with anammox contributing 61 % and denitrification contributing 39 %. Microbiological analyses indicated that the dominant microorganisms were *Candidatus Brocadia, Thauera, Denitratisoma*, and *Nitrosomonas*. In conclusion, study provides a solid foundation for the broader implementation of the SNAD process in wastewater treatment systems.

## Highlights

• Full-scale SNAD system successfully treated real low C/N wastewater.
• NRR was 0.9 kg/(m^3^·d) with anammox contributing 61 %.
• Rapid initiation of partial nitration with low DO and high FA.
• Organic matter was maximally resourced by heterotrophic bacteria.

## Graphical abstract

**Figure:**
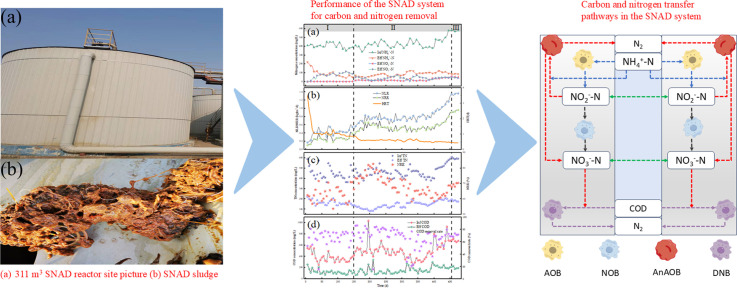

## 1 Introduction

The traditional treatment process for wastewater containing high levels of ammonia nitrogen and organic matter utilizes an internal circulation anaerobic reactor (IC) combined with anoxic/aerobic (A/O) processes (Fan et al., 2022). Due to the utilization of carbon resources in the IC, the wastewater entering the A/O system has the characteristics of high NH₄^+^–N, a low ratio of carbon to nitrogen (C/N), and relatively stable pollutant concentrations. Consequently, a large amount of additional organic carbon is necessary for nitrogen removal in A/O systems, leading to the inability of IC-A/O systems to achieve carbon source self-sufficiency (Shi et al., 2022). Furthermore, the A/O process involves the conversion of NH₄^+^–N to NO₃^−^–N, which necessitates substantial electrical energy input to provide sufficient aeration, thus conflicting with the energy-saving goal of wastewater treatment plants (WWTPs) (Adams et al., 2025). Additionally, sludge disposal remains a significant challenge that has raised widespread concern (Cao et al., 2017). Therefore, employing the conventional A/O process for the treatment of low C/N wastewater is becoming increasingly inefficient from both an economic and resource recovery standpoint.

Anaerobic ammonia oxidation (anammox) is a technology with great potential for nitrogen removal that has already shown advantages in treating high-ammonia wastewater (Jalili Jalalieh et al., 2024). Under anoxic conditions, NH₄^+^–N and NO₂^−^–N act as an electron donor and electron acceptor, respectively, reacting directly to produce nitrogen and NO₃^−^–N (Dong et al., 2024). Compared with the traditional IC-A/O process, the anammox process requires no carbon injection, needs a low level of aeration (40 %), and produces little sludge (10 %) (Chen et al., 2024). However, NH₄^+^–N and NO₂^−^–N are essential for anammox, and since the primary component of wastewater is NH₄^+^–N with minimal NO₂^−^–N, this presents a challenge. To overcome this limitation, the solution is to combine anammox with the partial nitrification process (PN-A), which utilizes ammonia-oxidizing bacteria (AOB) to produce NO_2_^−^–N (Yu et al., 2024). Nonetheless, PN-A is limited in its ability to treat NO_3_^−^–N and organic matter. Therefore, the simultaneous partial nitrification, anammox, and denitrification (SNAD) process is a better choice. The SNAD process removes nitrogen and carbon through controlling environmental conditions so that AOB, anammox bacteria (AnAOB), and denitrifying bacteria (DNB) can survive in a single reactor (Chen et al., 2009; Daverey et al., 2015). Unlike PN-A, the SNAD process can achieve the removal of organic matter and the small amount of NO₃^−^–N produced by anammox and nitrification processes, thereby achieving enhanced nitrogen removal (Singh et al., 2022). However, there are few successful examples of full-scale SNAD processes. This is mainly because the conditions of the SNAD process are extremely harsh (Singh et al., 2022). First, the supply of substrate NO_2_^−^–N necessary for anammox is unstable due to the difficulty of suppressing nitrite-oxidizing bacteria (NOB) (Du et al., 2021). The difference in oxygen half-saturation coefficients between AOB (0.32 mg/L) and NOB (0.70–5.30 mg/L) means that it is easier for AOB to obtain oxygen (Choi and Jung, 2023). Therefore, low dissolved oxygen strategies are often used to inhibit NOB. In addition, the growth of NOB can be inhibited by high free ammonia (FA) and high free nitrous acid (FNA) concentrations (Wang et al., 2022). Second, the suitable C/N for the SNAD process is relatively low, usually ranging from C/*N* = 3–4, and high concentrations of organic matter are not conducive to the enrichment of AnAOB (Singh et al., 2022). In addition, C/N fluctuations alter the ecological niches of AOB, NOB, AnAOB, and DNB, leading to system destabilization. Fortunately, the C/N of the IC effluent in the present study remained relatively stable at 1, avoiding this potential problem. Therefore, the main challenges in implementing the SNAD process are the inhibition of NOB, the enrichment of AnAOB and AOB, and synchronized denitrification involving DNB.

In this study, the IC effluent from the treatment of real amino-acid wastewater was utilized as the SNAD influent, providing a relatively suitable and stable C/N, and the direct start-up of the SNAD reactor was attempted. Furthermore, batch experiments and mass balance calculations were employed to analyze carbon and nitrogen degradation pathways, while high-throughput sequencing was conducted to examine the enrichment of microorganisms at different stages. The overall objective of this research was to develop a practical guidance program for the engineering application of the SNAD process.

## 2 Results and discussion

### 2.1 SNAD reactor startup process

At the beginning of the start-up period (0–50 d), the influent NH_4_^+^–N was controlled at 400 ± 15 mg/L and the temperature was maintained at 38 ± 1.5 °C. The effluent NH_4_^+^–N concentration was 100 mg/L, the effluent NO_3_^−^–N concentration increased rapidly to 100 mg/L, and the effluent NO_2_^−^–N concentration was 0 mg/L (Fig. 2a). The total nitrogen (TN) removal efficiency (NRE) of the system fluctuated around 60 %. The concentrations of both NH_4_^+^–N and NO_3_^−^–N were higher than expected due to two primary factors. First, the inoculated activated sludge was not domesticated, allowing the dominance of NOB, which resulted in a high NO_3_^−^–N concentration (Hausherr et al., 2022). Second, the inoculated sludge contained a large amount of organic matter, which led to the proliferation of DNB and competition with AOB for dissolved oxygen (DO), resulting in inefficient NH_4_^+^–N conversion (Li et al., 2017). These factors combined created an unfavorable for startup of the SNAD process. To address this issue, a short-lived high dissolved oxygen (DO) (0.5–2.0 mg/L) strategy was adopted to promote the rapid reduction of organic matter by DNB in the sludge and create a desirable low C/N environment.

**Fig. 1:**
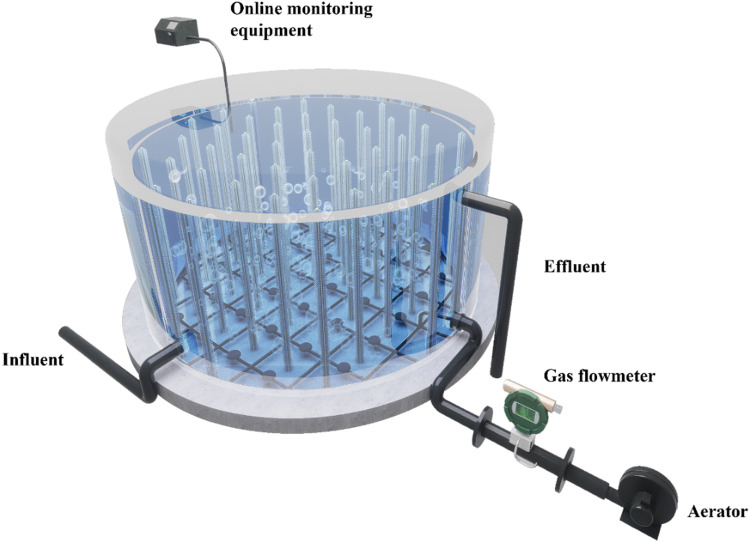
detailed overview of the interior of the SNAD reactor.

**Fig. 2:**
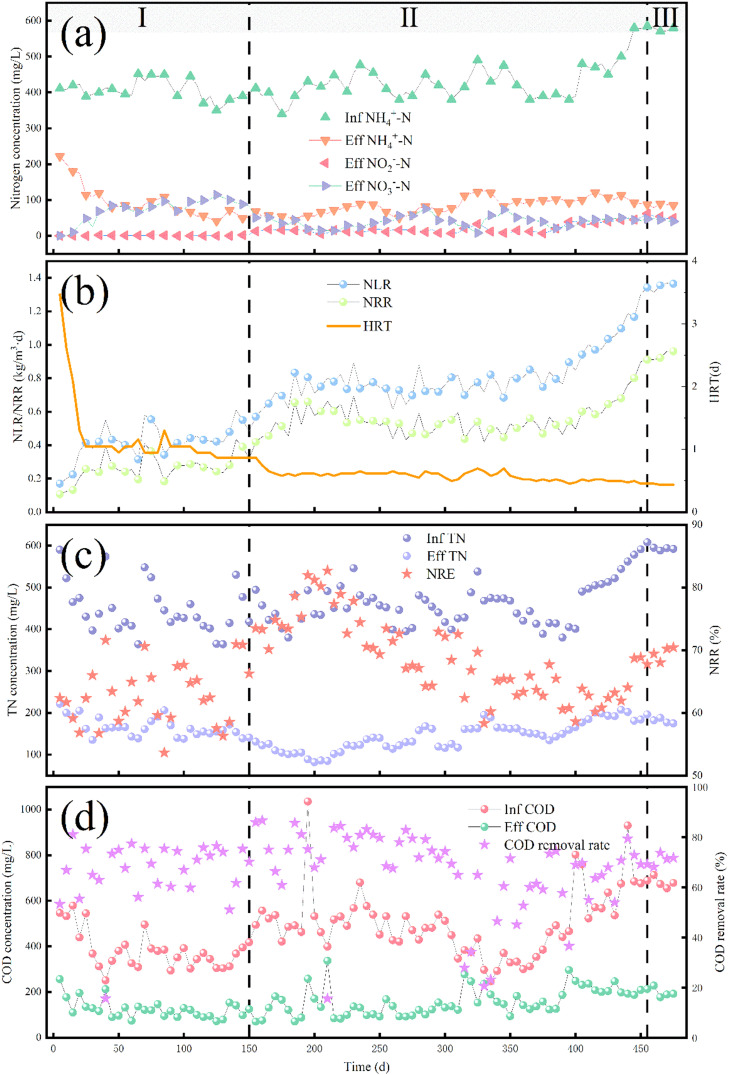
Characterization of the reactor operation: (a) changes in nitrogen; (b) changes in NLR/ NRR and HRT; (c) changes in TN and NRE; and (d) COD changes and removal rates.

**Fig. 3:**
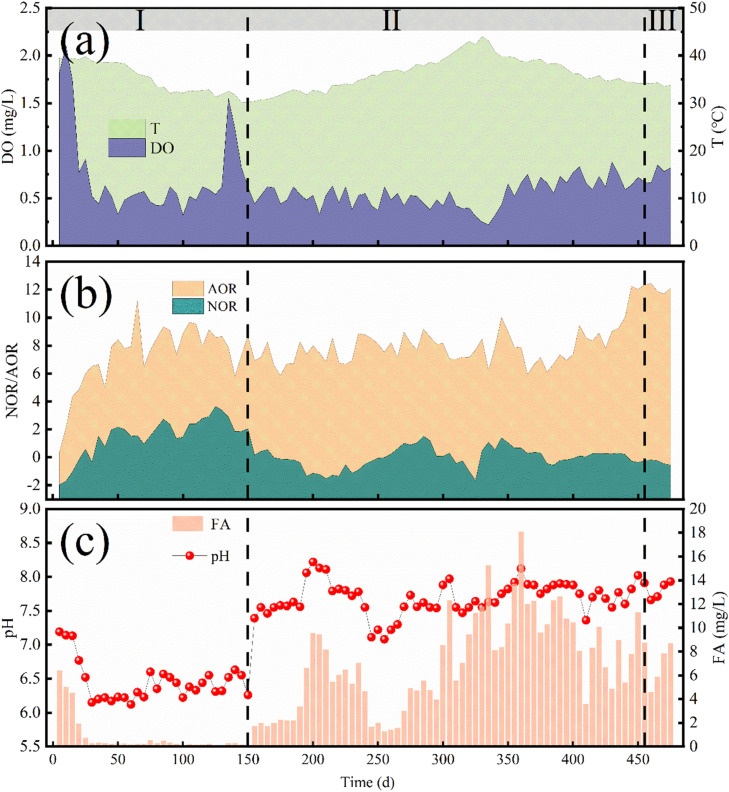
(a) Temperature and DO; (b) AOB(AOR)/ NOB(NOR) activity; and (c) FA and pH of the reactor.

At 50–150 d, the effluent conditions remained unchanged, and the start-up of the SNAD process progressed towards completion. During this period, low DO (approximately 0.5 mg/L) was maintained. By 150 d, the influent NH_4_^+^–N concentration was 390 mg/L, the effluent NH_4_^+^–N concentration was about 50 mg/L, and the effluent NO_3_^−^–N concentration had been reduced from 110 to 60 mg/L. The concentration of influent chemical oxygen demand (COD) was approximately 400 mg/L, while the concentration of effluent COD was approximately 100 mg/L. About 75 % of the organic matter was degraded by heterotrophic bacteria (Fig. 2d). The system's TN removal rate (NRR) was approximately 0.4 kg/(m^3^·d), much higher than that of conventional biological nitrogen removal, demonstrating the successful realization of the SNAD process (Yin et al., 2024). The regulation of low DO levels favors the inhibition of NOB, leading to the predominance of AOB (Li et al., 2021). The utilization of an appropriate FA concentration (about 5 mg/L) also played a key role. It has been shown that the upper tolerance limits of FA for AOB and NOB are 10–150 and 0.1–1.0 mg/L, respectively (Vadivelu et al., 2007). At this stage, AOB gradually became dominant, with the ammonia oxidation rate (AOR) and nitrite oxidation rate (NOR) reaching 8 mg/(L·h) and 0 mg/(L·h), respectively (Fig. 3b). It was noteworthy that a fixed carrier also contributed significantly to the successful start-up of the SNAD process. The addition of a fixed carrier enhances the retention of microorganisms with low growth rates, thereby improving their adaptability to fluctuating operational conditions, such as pollutant load and DO levels, as highlighted in previous studies (Kaewyai et al., 2022). The SNAD system in this study was successfully started within 150 d, which is relatively rapid compared with similar studies (Xu et al., 2018).

### 2.2 Increased pollutant loading and stabilized carbon and nitrogen removal

From 150 to 305 d, the influent NH_4_^+^–N was maintained at 400 mg/L and the influent COD was about 500 mg/L. To increase the pollutant load, the influent water quantity was raised. By 305 d, the NLR had increased to 0.8 kg/m^3^·d. The effluent NH_4_^+^–N, NO_2_^−^–N, and NO_3_^−^–N concentrations were 77, 7, and 44 mg/L, respectively, and the NRE was 70 % (Fig. 2a). The presence of residual NO_2_^−^–N in the effluent indicated the stabilization of partial nitrification. Due to the increase in influent water quantity and pH, the FA concentration rose to approximately 10 mg/L, which contributed to a more effective suppression of NOB. This was further evidenced by a more pronounced difference between the AOR and NOR of 7.04 and 0.30 mg/(L·h), respectively (Fig. 3b). At 445 d, the influent NH_4_^+^–N concentration was 580 mg/L, and the effluent NH_4_^+^–N, NO_2_^−^–N, and NO_3_^−^–N concentrations were 92, 53, and 44 mg/L, respectively, with an NRE of 67 %. Notably, between 200 and 330 d, the temperature of the reactor reached a peak of 43 °C due to the continuous increase in environmental temperature (Fig. 3a). This resulted in an increase in the NH_4_^+^–N concentration in the effluent to 200 mg/L, a decrease in the NRE to 34 %, and a decrease in the NRR to 0.34 kg/(m^3^·d). This decline was attributed to the inhibition of AOB activity caused by excessively high temperatures (Gabarró et al., 2012). The system returned to normal operation following a temporary reduction in aerator heat and the removal of the reactor cover.

The SNAD process operated stably between 445 and 475 d The NLR was maintained at 1.2 kg/(m^3^·d), and when the influent NH_4_^+^–N concentration was stabilized at about 570 mg/L, the effluent NH_4_^+^–N, NO_2_^−^–N, and NO_3_^−^–N concentrations were stabilized at about 85, 48, and 40 mg/L, respectively, with the NRE remaining 70 % (Fig. 2b). The COD removal efficiency was stabilized at about 70 %. It should be noted that the water quality did not meet the discharge standards after SNAD processing, and further deep nitrogen removal was required. Only 30 % of organics remained unremoved, which was mainly because these organics were not biodegradable. Not only does the SNAD process remove most of the organic matter in the influent, but the high nitrogen removal efficiency also ensures that the IC process can utilize additional organic matter in the raw amino acid wastewater to generate methane, thereby maximizing the resource utilization efficiency. This process did not add any organic matter, while maintaining a C/N ratio of about 1, and reached a very high NRR of up to 0.9 kg/(m^3^·d), which was much higher than that achieved using conventional biological treatment technology (Wu et al., 2024).

### 2.3 Microbial activity analysis

**Fig. 4:**
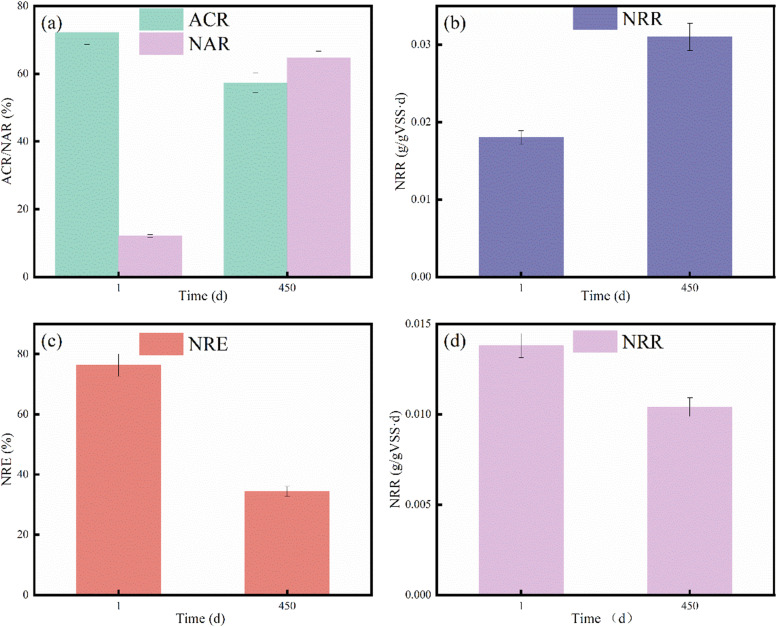
Analysis of sludge microbial activity in the SNAD system: (a) ACR/NAR of activated sludge; (b) NRR of AnAOB; (c) NRE of DNB; and (d) NRR of DNB.

At 450 d of stable reactor operation, sludge samples on the fixed carrier were extracted for batch experiments to determine the bacterial activity. Control samples of the original sludge were used for comparison. Under identical experimental conditions for activated sludge, the ammonia nitrogen conversion rate (ACR) and the nitrite accumulation rate (NAR) shifted from 77.2 % and 12.1 % at startup, respectively, to 57.3 % and 64.7 % at 450 d, respectively (Fig. 4). This demonstrated the successful realization of partial nitrification with the stable production capacity of NO_2_^−^–N. For AnAOB, the NRR increased from 0.018 g/(gVSS·d) at initiation to 0.031 g/(gVSS·d) at stabilization, indicating that the nitrogen removal ability of the system was nearly tripled. This suggests that the AnAOB were well adapted to full-scale application environments. Although excessive organic matter may inhibit AnAOB activity, supplying the appropriate amount of organic matter can provide microorganisms with the elements necessary for growth, promote microbial metabolism, and accelerate microbial growth (Liu et al., 2024). Practical results indicate that a C/N ratio of 1 or less is optimal AnAOB enrichment. The NO_3_^−^–N removal efficiency of DNB decreased from 83.3 % to 78.4 %, while the NRR decreased from 0.0138 to 0.0104 g/(gVSS·d). There was a slight reduction in the nitrogen removal capacity of DNB, which was to be expected. DNB utilize nitrate and carbon sources to remove nitrogen. Due to the long-term unfavorable environment of low C/N in the influent, the associated DNB were unable to adapt, resulting in a decline in treatment efficiency. The DNB were gradually disadvantaged in competition with AnAOB on substrates. However, this portion of the DNB was still critical to the improvement of the NRR. AnAOB and DNB collaborated in the SNAD system to jointly achieve high TN removal efficiency. The specific contribution rates are provided in Section 2.4.

### 2.4 Chemometric analysis of carbon and nitrogen transfer

The nitrogen removal process within the SNAD system was primarily driven by anammox and denitrification. Moreover, most of the COD in the SNAD system was removed through denitrification, while a small fraction was removed by other heterotrophic bacteria. To better understand the contribution of each functional bacterial group to nitrogen removal, mass balance calculations of the SNAD process were combined with batch experiment results, enabling a more precise determination of the nitrogen removal capacity of each bacterial genus (Wang et al., 2010).

The analysis was based on several assumptions: (1) partial nitrification was followed by anammox and denitrification; (2) during anammox, the ratio of NH_4_^+^–N to NO_2_^−^–N was 1.32, with 1 mol NH_4_^+^–N reacting to produce 0.26 mol NO_3_^−^–N; and (3) 1 mg NO_3_^−^–N consumed 2.87 mg of COD.

**Fig. 5:**
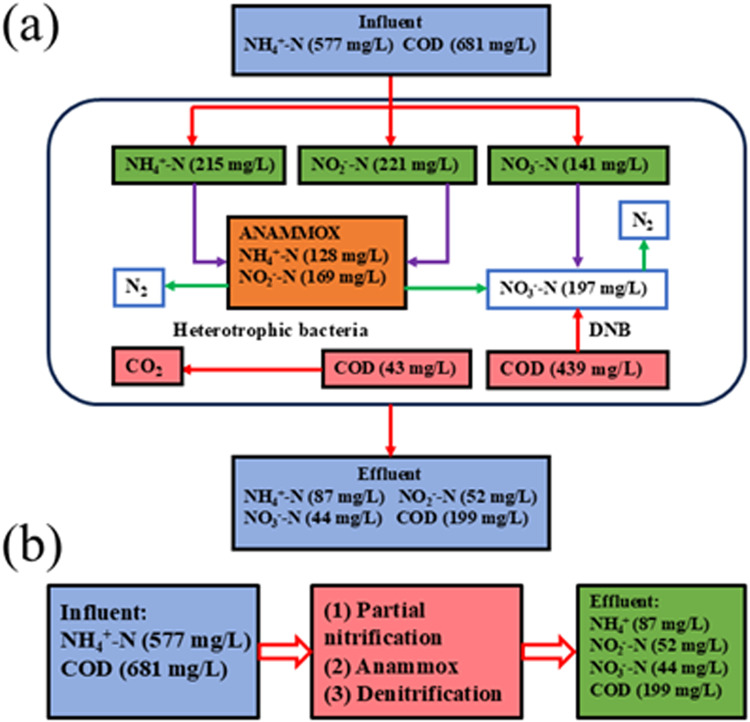
(a) Carbon and nitrogen transfer pathways and (b) overall performance of the SNAD system.

When the concentrations of influent NH_4_^+^–N and COD were 577 and 618 mg/L, respectively, about 38 % and 24 % of NH_4_^+^–N were converted into NO_2_^−^–N and NO_3_^−^–N, respectively (Fig. 5). This conversion rate was higher than expected based on the batch experiments, possibly because the pH did not reach the optimal conditions for partial nitrification during the actual operation. Moreover, the contributions of anammox and denitrification to nitrogen removal were 61 % and 39 %, respectively. These findings highlight that anammox was the dominant contributor to nitrogen removal in the SNAD system, while denitrification played a secondary role. For COD removal, 44 % of COD was used for denitrification, 9 % was utilized by other heterotrophic bacteria, and 45 % was resistant to biodegrade. This higher proportion of difficult-to-biodegrade COD compared to actual site operation may be attributed to the consumption of organic matter by other heterotrophic bacteria during the site operation. Overall, these results indicate that partial nitrification, anammox, and denitrification occurred simultaneously in the reactor. The synergistic effect of partial nitrification and anammox was the primary driver of nitrogen removal, and denitrification serving as a secondary contributor.

### 2.5 High-throughput sequencing to analyze microbial diversity

**Fig. 6:**
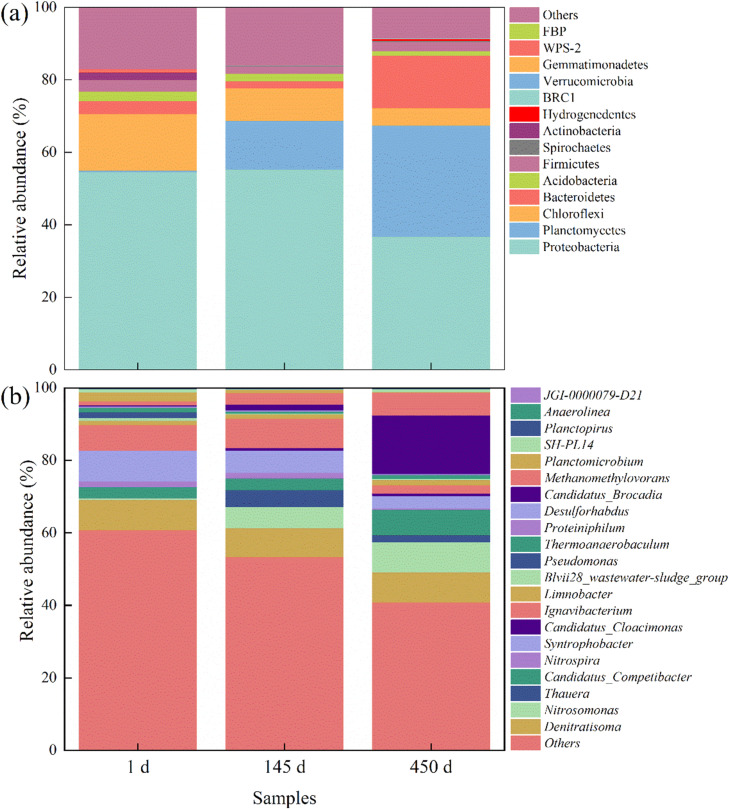
Relative abundances of microorganisms at the (a) phylum and (b) genus levels.

The relative abundance of microorganisms at the phylum level is shown in Fig. 6a, where the most abundant phyla include Proteobacteri, Chloroflexi, Bacteroidetes, Firmicutes, Planctomycetes, and Actinobacteria. Among these phyla, the primary microorganisms involved in nitrogen removal were Proteobacteria and Planctomycetes. Proteobacteria constituted the main group of bacteria present during wastewater treatment, and most of these microorganisms were associated with biological nitrogen removal. Notable nitrification bacteria, such as *Nitrosomonas* and *Nitrosococcus* and other common AOB and NOB such as *Nitrotoga, Nitrobacter, Nitrococcus*, and DNB all belong to this phylum (Zhang et al., 2012). All species of functional AnAOB bacteria belonged to phylum Planctomycetes. Because the sludge in the system was inoculated from the return sludge of the secondary sedimentation reactor with low anammox activity, which contained large amounts of AOB and NOB. As a result, the relative abundance of Proteobacteria was higher (54.66 %), while the relative abundance of Planctomycetes was lower (0.35 %). When the NLR was 1.2 kg/(m^3^·d) during the stable period, the relative abundances of Proteobacteria and Planctomycetes were 30.1 % and 34.1 %, respectively, resulting in a total occupancy of 64.2 %, and the NRE reached 70 % at this time. The substantial enhancement of Planctomycetes may imply the successful enrichment of AnAOB. Chloroflexi is a parthenogenetic anaerobic microorganism that is commonly found in anaerobic reactors, and the presence of such bacteria is often observed in anammox reactors, contributing to the formation of AnAOB and the degradation of sugars and some compounds (Kindaichi et al., 2012). Interestingly, the relative abundance of Chloroflexi increased rather than decreased when the DO was elevated in the late system, increasing from 5 % at 1 d to 11 % at 450 d Bacteroidetes is also a highly variable phylum; this group of bacteria has a filamentous structure that favors biofilm formation, with a relative abundance of 14 % at 450 d The variation may have been related to the fixed carrier, which gradually became thicker as the system's denitrification capacity increased (Guo and Zhang, 2012). Actinobacteria, most of which are aerobic, are frequently found in common activated sludge. The relative abundance of Actinobacteria decreased more significantly in the oxygen-limited SNAD system, declining from an initial 2.61 % to 1.25 % (Wang and Li, 2023).

Changes in the structural genera of the microbial community are depicted in Fig. 6b. *Candidatus Brocadia* was the only AnAOB found in the SNAD. *Candidatus Brocadia* exhibited a significant competitive advantage in the presence of organic matter, which was consistent with the results of related studies. The relative abundance of *Candidatus Brocadia* increased from 0.32 % at the beginning of the startup to 1.56 % at 150 d During the stabilization period, the relative abundance of *Candidatus Brocadia* increased to 16.3 %, the NRR of the system reached 1.2 kg/(m^3^·d), and the TN removal could reach 70 %, indicating that *Candidatus Brocadia* played critical role in nitrogen removal. *Nitrosomonas*, the only AOB found in the SNAD system, was frequently detected in sludge from different SNAD reactors (Zhou et al., 2024). Its relative abundance increased from 0.55 % at the beginning of startup to 8.2 %, reflecting the increasing shortcut nitrification capacity of the system. *Nitrospira*, an NOB, was also involved in nitrogen conversion in the SNAD reactor. The findings demonstrated that inhibiting NOB activity during the startup of the SNAD reactor was critical for the initiation of the anammox process. The relative abundance of *Nitrospira* was 1.55 % in the initial inoculated sludge and decreased to 0.38 % at 450 d, indicating that NOB were eliminated at this time. The increase in the relative abundance of *Nitrosomonas* and the decrease in *Nitrospira* were intrinsic to the enhanced partial nitrification. Meanwhile, the SNAD system exhibited an increasing capacity for nitrogen removal, with AnAOB contributing the most to nitrogen removal, while DNB accounted for a significant proportion. The denitrifying genera detected during the late startup period were *Thauera* and *Denitratisoma*, with relative abundances of 3 % and 3.2 %, respectively. Some studies have suggested that the SNAD process can achieve efficient synergistic nitrogen removal in the reactor, if the C/N of the influent is properly controlled (Wang et al., 2018). The decrease in abundance of *Thauera* was more pronounced (from 4.66 % to 1.86 %), while the change in the relative abundance of *Denitratisoma* was smaller (8.30 % to 8.32 %). The lower concentration of organic matter during operation was not favorable for DNB propagation. Among the DNB, *Denitratisoma* maintained a relatively stable denitrification effect, while *Thauera* decreased, possibly due to an inability to tolerate the semi-autotrophic environment, although *Thauera* still contributed to the denitrification of the system. The abundances of *Ignavibacterium* (7.13 %–2.28 %) and *Syntrophobacter* (8.41 %–3.38 %) changed significantly during the reactor operation. As a non-photosynthetic nutrient bacterium, *Ignavibacterium* can survive in both anoxic and aerobic environments and plays an important role in anammox. The increasing abundance of *Ignavibacterium* detected in the present study was in line with the trend of the system's ability to remove nitrogen (Wang et al., 2016). It is possible that the decreased relative abundance of *Ignavibacterium* was related to the increase in the relative abundances of AnAOB and other bacteria. *Syntrophobacter* is usually detected in anaerobic digesters, in which it plays an important role in the degradation of propionic acid. Its detection in the SNAD reactor may have been due to the input of a small amount of sludge from the IC system into the SNAD reactor (Usman et al., 2023). The data from the combined microbial diversity assay confirmed that the system's ability to remove nitrogen was derived from the synergistic effect of AOB, AnAOB, and DNB.

## 3 Conclusion

This study implemented a full-scale SNAD system for the treatment of the IC effluent of amino acid wastewater to enhance the efficiency of organic matter utilization. After 150 d, the SNAD process was successfully initiated by regulating FA (0.8 ± 0.3 mg/L) and DO (2–12 mg/L). During the stabilization period, the removal of nitrogen and carbon reached 70 % and 75 %, respectively, with an NRR of 0.9 kg/(m^3^·d). Batch experiments and chemometric analysis further demonstrated synergistic nitrogen removal by anammox (61 %) and denitrification (39 %). The dominant microorganisms in the system were *Candidatus Brocadia, Thauera, Denitratisoma*, and *Nitrosomonas*. Overall, this study provides valuable insights for the engineering application of SNAD.

## 4 Materials and methods

### 4.1 Reaction devices and development procedures

A continuous flow reactor was employed in the present study. The reactor was composed of high-purity aluminum, with an overall volume of 311 m^3^. A fixed carrier was installed for sludge retention and preservation, and a small amount of suspended sludge went into the settling reactor at the back. Aeration was supplied to the reactor through an aerator, which also promoted sludge–water mixing. To mitigate heat loss, the exterior of the reactor was insulated with foam. A cover was installed on the top of the reactor to avoid the influence of light (Fig. 1).

The overall operational sequence can be separated into three distinct phases: the start-up period (I), the pollutant load enhancement period (II), and the stable operation period (III). The increase in pollutant loading was achieved by increasing the water flow and thus reducing the hydraulic retention time (HRT). The temperature of the reaction was mainly determined by the water temperature, and no heating device was installed. To achieve partial nitrification, the reactor was operated with lower DO content. Detailed operational parameters are provided in Table 1.

**Table 1:** Reactor operating parameters.

Stage	Time (d)	DO (mg/L)	pH	HRT (d)	Temperature ( °C)
I	0–150	0.42–2.00	6.10–7.20	3.50–0.87	30–39
II	150–455	0.22–0.80	7.40–8.20	0.45–0.87	30–43
III	455–475	0.65–0.85	7.60–8.00	0.43–0.45	33–34

### 4.2 Sludge inoculation and pollutant information

The original sludge was procured from the secondary sedimentation reactor of an A/O system in Qinghuangdao, China. This A/O process has been in stable operation for more than 600 d with an influent TN loading (NLR) of 0.12 kg/m^3^·d. The sludge was a flocculated nitrified sludge with an initial mixed liquid suspended solids (MLSS) content of 4000 mg/L. Due to the excellent performance of the sludge, it grew on the fixed carrier in a short time, while the remaining sludge flowed out of the reactor with the effluent. Therefore, the reaction was mainly conducted by the microorganisms on the fixed carrier, with the contribution from suspended sludge being negligible.

The wastewater introduced into the reactor originated from the effluent of a bioproduct production facility in Qinhuangdao, China. As a result of the IC treatment, the wastewater contained low levels of biodegradable organic matter, with a C/N ratio of <1. The primary form of nitrogen present was NH_4_^+^–N. Moreover, the wastewater was free of toxic heavy metals and did not inhibit microbial activity. Detailed information on the water quality is provided in Table 2.

**Table 2:** Detailed water quality parameters.

Parameters	COD (mg/L)	TN (mg/L)	NH_4_^+^–N (mg/L)	Quantity (m^3^)
	300–600	400–600	400–600	280–600

### 4.3 Batch test for functional microbial activity

To determine the maximum activities of AOB, AnAOB, and DNB, activated sludge from the fixed carrier was collected for batch experiments during the stable operational period. The sludge was placed into a 1-L beaker, ultrapure water was added, and the mixture was stirred well. The supernatant was poured out after the sludge was allowed to settle. This washing procedure was repeated three times to remove the residual substrates from the sludge. Subsequently, the sludge was transferred to a 500-ml conical flask equipped with a stirring device. The level of mixed liquid volatile suspended solids (MLVSS) was adjusted to 3000 mg/L, and the concentrations of NH_4_^+^–N, NO_2_^−^–N, and NO_3_^−^–N were set at 50 mg/L. For the DNB experiments, COD was prepared from sodium acetate at a concentration of 250 mg/L. The sources of NH_4_^+^–N, NO_2_^−^–N, and NO_3_^−^–N were NH_4_HCO_3_, NaNO_2_, and NaNO_3_, respectively; industrial-grade sodium bicarbonate provided the inorganic carbon source and alkalinity; and KH_2_PO_4_ (25.2 mg/L), CaCl_2_·2H_2_O (200 mg/L), and MgSO_4_·7H_2_O (280 mg/L) were injected. The concentrations of trace elements I and II were 2.0 and 2.4 mg/L, respectively, and the formulations of these trace elements were consistent with those described by Yu et al. (Yu et al., 2024). For the AOB experiment, aeration was provided, with conditions designed to closely replicate the actual SNAD system, maintaining a DO of 0.65 mg/L. Water samples were collected at one-hour intervals in a 30 °C shaker to analyze the nitrogen composition. The experiment was conducted with three replicates. Detailed information on the experiment setup is displayed in Table 3.

**Table 3:** Setting information for batch experiments.

Batch test	DO	NH_4_^+^–N	NO_2_^−^–N	NO_3_^−^–N	COD
AOB	0.65 mg/L	50 mg/L	–	–	–
AnAOB	–	50 mg/L	50 mg/L	–	–
DNB	–	–	–	50 mg/L	250 mg/L

### 4.4 Routine test

Daily testing for NH_4_^+^–N, NO_2_^−^–N, NO_3_^−^–N, COD, and TN was performed based on established national standard methods (Rice et al., 2012). The MLSS content of activated sludge was determined using the gravimetric method. The DO, pH, and temperature data were obtained using online monitoring equipment (WTW340i, Germany).

### 4.5 Biological experimental methods

Sludge samples were collected from the reactor at 1, 145, and 450 d for the analysis of microbial diversity. The samples were stored in *a* − 80 °C refrigerator and subsequently dispatched to Majorbio in Shanghai, China, for analysis. The FastDNA™ Spin Kit (Shenergy Biocolor, China) was employed to extract DNA from the samples according to the manufacturer's instructions. The extracted genomic DNA was detected using 1 % agarose gel electrophoresis. High-throughput amplification sequencing of DNA sequences from the V3–V4 region of the 16S rDNA gene was conducted using the bacterial universal primers 338F (ACTCCTACGGGAAGCA) and 806R (GGACTACHVGGGTWTCTAAT) (Xu et al., 2024). Following amplification using a polymerase chain reaction (PCR) instrument (ABI GeneAmp® Model 9700), sequencing was performed on the Illumina platform.

### 4.6 Calculation method

The relevant equations are shown as follows. The FA levels were calculated with reference to the Anthonisen balance equation (Anthonisen et al., 1976). The equations used to calculate the activity of AOB (AOR) and NOB (NOR) were obtained based on the nitrogen balance and theoretical stoichiometry (Yue et al., 2018).

$$NLR=\frac{TN_{in}}{HRT}.$$

$$FA=\frac{17}{14}\times \frac{\left[NH_{4}^{+}-N\right]\times 10^{pH}}{e^{\frac{634}{273+T}}}+10^{pH}.$$

$$AOR=\frac{\Delta NH_{4}^{+}-N-\frac{\Delta TN}{2.04}}{24}.$$

$$NOR=\frac{\Delta NO_{3}^{-}-N-\frac{\Delta TN}{2.04}\times \;0.26}{24}.$$

$$NRE=\frac{TN_{in}-TN_{eff}}{TN_{in}}\times \;100\%.$$

(1) $$NRR=\frac{TN_{in}-TN_{eff}}{HRT}$$

(2) $$NRR=\frac{\Delta TN}{MVLSS\cdot d}\cdot$$

$$ACR=\frac{100\cdot \Delta \left[NH_{4}^{+}-N\right]}{NH_{4}^{+}-N}\cdot$$

$$NAR=\frac{100\cdot [NO_{2}^{-}-N]}{[NO_{2}^{-}-N]+[NO_{3}^{-}-N]},$$

where TN_in_ and TN_eff_ refer to the TN concentrations in the influent and effluent of the SNAD reactor, respectively. HRT is the hydraulic retention time of the reactor, expressed in days. The unit of AOR/NOR is mg/(L·h). The NRR (1) equation is used for the reactor, with units of kg/(m^3^·d), and the NRR (2) equation is used for the batch experiment, with units of g/gVSS·d.

## CRediT authorship contribution statement

**Xi Cao:** Writing – review & editing, Formal analysis, Conceptualization. **Tianqi Liu:** Writing – review & editing, Formal analysis, Conceptualization. **Xiang Li:** Resources, Formal analysis, Conceptualization. **Yong Huang:** Resources. **Qin Nie:** Formal analysis, Data curation. **Ming Li:** Formal analysis.

## Declaration of competing interest

The authors declare that they have no known competing financial interests or personal relationships that could have appeared to influence the work reported in this paper.

## Data Availability

Data will be made available on request
